# Influenza Vaccination of Swine Reduces Public Health Risk at the Swine-Human Interface

**DOI:** 10.1128/mSphere.01170-20

**Published:** 2021-06-30

**Authors:** Joshua N. Lorbach, Sarah W. Nelson, Sarah E. Lauterbach, Jacqueline M. Nolting, Eben Kenah, Dillon S. McBride, Marie R. Culhane, Christa Goodell, Andrew S. Bowman

**Affiliations:** a The Ohio State University, Columbus, Ohio, USA; b University of Minnesota, St. Paul, Minnesota, USA; c Boehringer Ingelheim Vetmedica, Inc., Duluth, Georgia, USA; University Medical Center Freiburg

**Keywords:** disease transmission, infectious, infectious disease, influenza A virus, preventive medicine, swine, transmission, vaccines, zoonoses

## Abstract

Influenza A viruses (IAV) in swine (IAV-S) pose serious risk to public health through spillover at the human-animal interface. Continued zoonotic transmission increases the likelihood novel IAV-S capable of causing the next influenza pandemic will emerge from this animal reservoir. Because current mitigation strategies are insufficient to prevent IAV zoonosis, we investigated the ability of swine vaccination to decrease IAV-S zoonotic transmission risk. We assessed postchallenge viral shedding in market-age swine vaccinated with either live-attenuated influenza virus (LAIV), killed influenza virus (KV), or sham vaccine (NV). We also assessed postchallenge transmission by exposing naive ferrets to pigs with contact types reflective of those experienced by humans in a field setting. LAIV and KV swine groups exhibited a nearly 100-fold reduction in peak nasal titer (LAIV mean, 4.55 log 50% tissue culture infectious dose [TCID_50_]/ml; KV mean, 4.53 log TCID_50_/ml) compared to NV swine (mean, 6.40 log TCID_50_/ml). Air sampling during the postchallenge period revealed decreased cumulative IAV in LAIV and KV study room air (LAIV, area under the concentration-time curve [AUC] of 57.55; KV, AUC = 24.29) compared to the NV study room (AUC = 86.92). Pairwise survival analysis revealed a significant delay in onset of infection among ferrets exposed to LAIV pigs versus NV pigs (rate ratio, 0.66; *P* = 0.028). Ferrets exposed to vaccinated pigs had lower cumulative virus titers in nasal wash samples (LAIV versus NV, *P* < 0.0001; KV versus NV, *P*= 0.3490) and experienced reduced clinical signs during infection. Our findings support the implementation of preexhibition influenza vaccination of swine to reduce the public health risk posed by IAV-S at agricultural exhibitions.

**IMPORTANCE** Swine exhibited at agricultural fairs in North America have been the source of repeated zoonotic influenza A virus transmission, which creates a pathway for influenza pandemic emergence. We investigated the effect of using either live-attenuated influenza virus or killed influenza virus vaccines as prefair influenza vaccination of swine on zoonotic influenza transmission risk. Ferrets were exposed to the pigs in order to simulate human exposure in a field setting. We observed reductions in influenza A virus shedding in both groups of vaccinated pigs as well as the corresponding ferret exposure groups, indicating vaccination improved outcomes on both sides of the interface. There was also significant delay in onset of infection among ferrets that were exposed to live-attenuated virus-vaccinated pigs, which might be beneficial during longer fairs. Our findings indicate that policies mandating influenza vaccination of swine before fairs, while not currently common, would reduce the public health risk posed by influenza zoonosis.

## INTRODUCTION

Interspecies transmission of influenza A viruses (IAV) represents a crucial pathway for emergence of novel IAVs that can significantly affect public health in the form of influenza outbreaks, epidemics, and pandemics. Prolonged contact between swine and humans at agricultural exhibitions in the United States and Mexico has been associated with hundreds of cases of human infection with swine-origin IAV (IAV-S), which makes fairs an important swine-human interface in terms of zoonotic transmission (1, 2). Bidirectional spillover events at this interface can have immediate and long-term effects on both human and animal health.

Given the repeated zoonotic IAV transmission that has occurred at agricultural exhibitions, or fairs, evidence-based mitigation strategies are needed to prevent IAV transmission between swine and humans. Agricultural fairs are unique swine concentration points because pigs with varied IAV exposure histories travel from distant localities to participate, allowing a few infected pigs to potentially infect a large number of immunologically naive pigs (3). Increased IAV prevalence in pigs at a fair increases the likelihood that susceptible humans coming into contact with swine at fairs will become infected with zoonotic influenza (4). Therefore, many strategies designed to mitigate the risk posed by IAV-S at the human-animal interface are focused on reducing the prevalence of IAV in swine (5).

Influenza is frequently subclinical in swine, making it nearly impossible to prevent entry of IAV-infected pigs into exhibitions (6). Animal and public health officials recommend shortening swine exhibitions to limit IAV amplification in swine and reduce the opportunity for spillover to humans (5, 7). The Centers for Disease Control and Prevention recommend individuals involved with swine shows receive annual seasonal influenza vaccinations, although this is primarily aimed toward prevention of transmission of seasonal IAVs from people to swine (8). Additional approaches to minimizing risk posed by IAV-S include educating the public who attend swine shows with signage that indicates young children, pregnant women, elderly individuals, and those with immunologic or cardiovascular health conditions may be at increased risk of infection or severe complications associated with influenza (9). Other prevention strategies include promoting personal hygiene by placing handwashing stations in the vicinity and separating food service areas from swine barns (5).

Pre-exhibition influenza vaccination of pigs has been considered a method to reduce the prevalence of IAV in exhibition swine. Exhibitors are advised to consult a veterinarian about administering influenza vaccines, but vaccination of pigs is not required at all exhibitions (5). Multiple IAV vaccine products are licensed for use in swine, but no studies have assessed the ability of these products to protect public health at the swine-human interface (10, 11). We sought to validate the implementation of swine influenza vaccination as a mitigation strategy to reduce the risk of spillover of IAV-S from swine to humans. We hypothesized that vaccination of swine could reduce the public health threat posed by IAV-S. To assess this, we compared the effect swine vaccination had on postchallenge viral shedding and subsequent transmission to naive ferrets in near exposure (NE) or far exposure (FE) using a translational study designed to simulate contact between IAV-infected swine and humans at agricultural exhibitions.

## RESULTS

### Swine virus infection and shedding.

**FIG 1:**
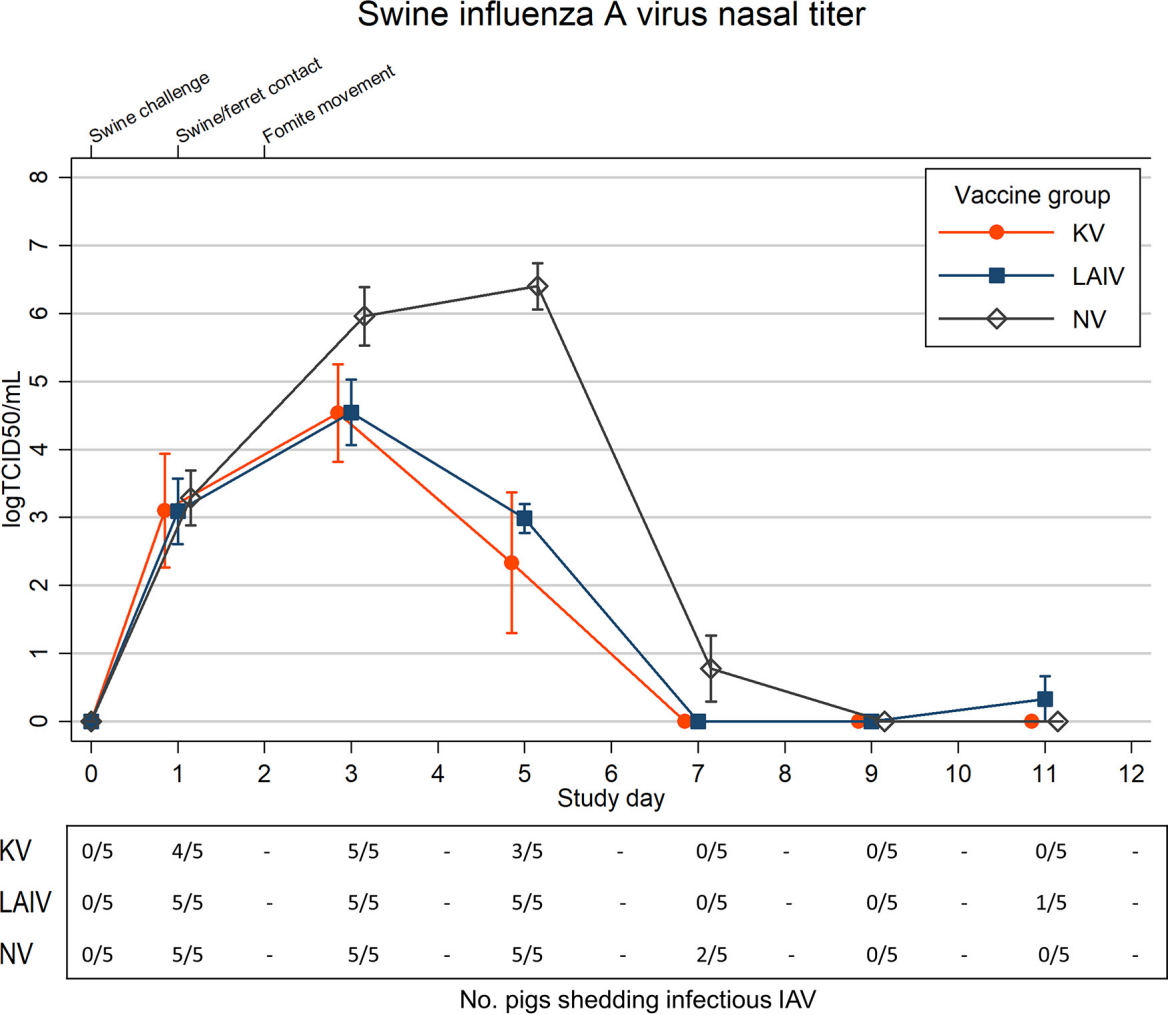
Mean (*n* = 5) swine nasal swab influenza A virus (IAV) titers are shown by vaccination group: live-attenuated influenza virus (LAIV), killed influenza virus (KV), or sham vaccine (NV). *N* = 5 pigs per group. Spikes indicate standard errors of the means (SEM). The lower *x* axis indicates individual study days, and values are skewed (±0.1) to allow comparison of overlapping error bars. The upper *x* axis denotes timeline points of interest. Fomite transfer occurred between pigs and near-exposed (NE) ferrets only. The corresponding table below line plot indicates number of animals shedding infectious virus, defined as an individual titer in excess of the assay limit of detection (LOD; 1.5 log TCID_50_/ml) by study day. Titers at or below LOD were assigned a nominal value of 0.0 for mean calculations.

All study swine were IAV positive by PCR 1 day postchallenge (dpc). Peak nasal swab titers were nearly 100-fold lower in live-attenuated influenza virus (LAIV)-vaccinated pigs than in sham-vaccinated (NV) pigs. Cumulative nasal virus shedding among swine in the LAIV and killed influenza virus (KV) groups were significantly reduced compared to the NV group, and comparison of LAIV and KV pigs revealed similar cumulative nasal shedding (see Table S1 in the supplemental material). Viral shedding peaked at 5 dpc in NV swine, whereas peak shedding occurred at 3 dpc for LAIV and KV swine (Fig. 1 and Table S2). Infectious IAV shedding ceased by 7 dpc for LAIV and KV pigs and by 9 dpc for NV pigs. No LAIV pigs were shedding infectious virus at 9 dpc; however, a single LAIV pig was isolation positive at 11 dpc.

10.1128/mSphere.01170-20.1 TABLE S1 Comparisons of within-group AUC estimates of cumulative IAV shedding in swine and ferrets. Download Table S1, DOCX file, 0.02 MB. Copyright © 2021 Lorbach et al. 2021 Lorbach et al. https://creativecommons.org/licenses/by/4.0/ This content is distributed under the terms of the Creative Commons Attribution 4.0 International license.

10.1128/mSphere.01170-20.2 TABLE S2 Mean influenza A virus titers (TCID_50_/ml) in swine nasal swab and ferret nasal wash samples. Download Table S2, DOCX file, 0.02 MB. Copyright © 2021 Lorbach et al. 2021 Lorbach et al. https://creativecommons.org/licenses/by/4.0/ This content is distributed under the terms of the Creative Commons Attribution 4.0 International license.

Three NV pigs experienced increases in respiratory rate above 40 breaths per minute following challenge. No clinical signs indicating respiratory distress were appreciated in LAIV or KV pigs. Lethargy was noted in all NV pigs following challenge, with one or more lethargic pigs observed between 4 and 10 dpc. In comparison, lethargy was observed in all KV pigs at 4 dpc and in one LAIV pig at 2 dpc.

### Swine-to-ferret transmission.

**FIG 2:**
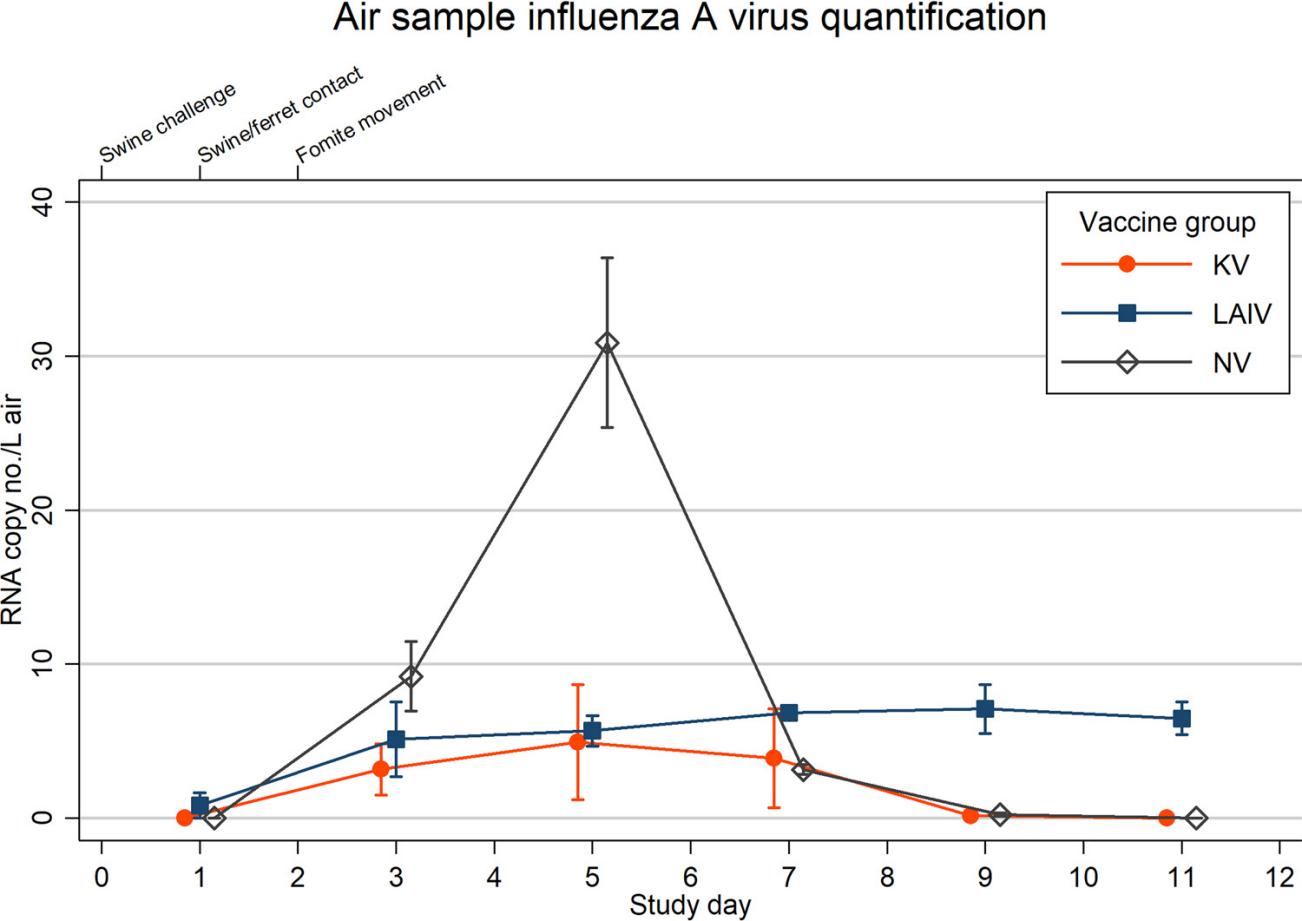
Mean RNA copy number per liter of air is shown by vaccination group: live-attenuated influenza virus (LAIV), killed influenza virus (KV), or sham vaccine (NV). *N* = 3 air sample devices for each group time point. Spikes indicate standard errors of the means (SEM). The lower *x* axis indicates individual study days, and values are skewed (±0.1) to allow comparison of overlapping error bars. The upper *x* axis denotes timeline points of interest.

The cumulative IAV genome copy number per liter of air was lower for the LAIV and KV groups than the NV group. IAV air levels for the NV group rose sharply before peaking at 5 dpc, corresponding with peak nasal shedding in NV swine (Fig. 1 and 2). Peak IAV air levels for the KV swine also occurred at 5 dpc, whereas peak levels for the LAIV swine occurred at 9 dpc.

**FIG 3:**
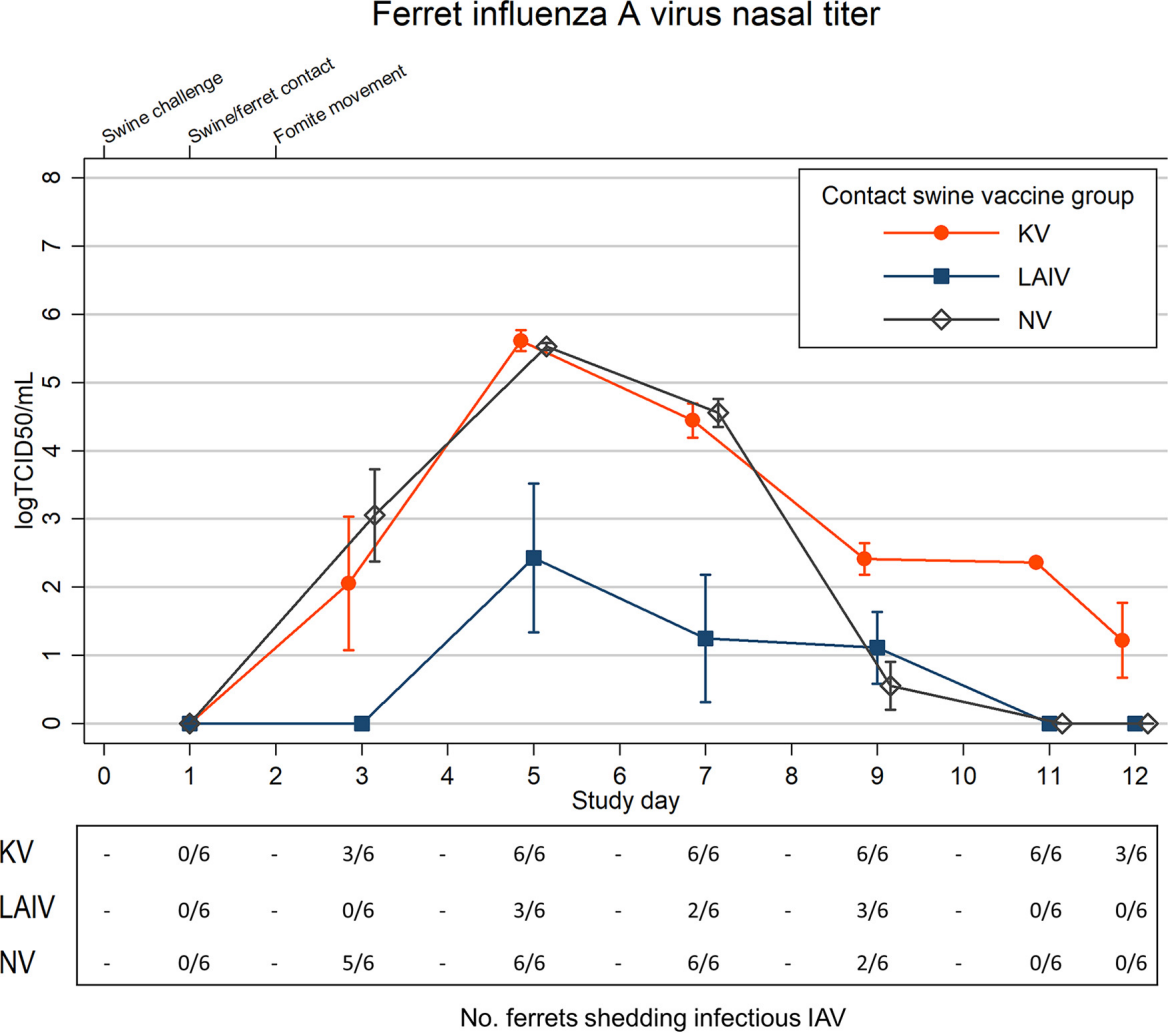
Mean (*n* = 6) ferret nasal wash influenza A virus (IAV) titers are shown by swine group exposure: live-attenuated influenza virus (LAIV), killed influenza virus (KV), or sham vaccine (NV). *N* = 6 ferrets per group. Titers at or below the assay limit of detection (LOD; 1.5 log TCID_50_/ml) were assigned a nominal value of 0.0 for mean calculations. Individual ferret nasal titer data are shown separately in the supplemental material (Fig. S3). Spikes indicate standard errors of the means (SEM). The lower *x* axis indicates individual study days, and values are skewed (±0.1) to allow comparison of overlapping error bars. The upper *x* axis denotes timeline points of interest. Fomite transfer occurred between pigs and near-exposed (NE) ferrets only. The corresponding table below the line plot indicates the number of animals shedding infectious virus, defined as an individual titer in excess of the assay LOD by study day.

All ferrets in the KV and NV groups were IAV positive by PCR at 2 days postexposure (dpe); infectious virus was recovered from five of six ferrets in the NV group and all three of the NE ferrets in the KV group. In contrast, while five of six ferrets in the LAIV group were PCR positive at 2 dpe, we did not recover any infectious IAV (Fig. 3 and Fig. S1). Infectious IAV recovery was delayed until 4 dpe for NE and FE ferrets in the LAIV group and for FE ferrets in the KV group. Overall, mean nasal shedding in all ferret groups peaked at 4 dpe (Fig. 3 and Fig. S1, Table S2). When further examined by exposure type, peak nasal shedding for FE ferrets in the LAIV group was delayed until 8 dpe (Fig. S1). There was one NE ferret in the LAIV group whose nasal wash viral titers were consistently below the assay LOD despite PCR-confirmed exposure to virus.

10.1128/mSphere.01170-20.3 FIG S1 Mean (*n* = 3) near-exposed (A), mean (*n* = 3) far-exposed (B), and all (*n* = 6) individual ferret (C) nasal wash influenza A virus (IAV) titers are shown by swine vaccination group exposure. (Inset) Live-attenuated influenza virus (LAIV), killed influenza virus (KV), or sham vaccine (NV). The lower *x* axis indicates individual study days, and the upper *x* axis denotes timeline points of interest, including intranasal IAV challenge of swine on day 0, introduction of naïve ferrets to study room on day 1, and fomite transfer between pigs and near-exposed ferrets on day 2. Individual markers are jittered to allow comparison of overlapping data. Titers at or below the LOD (1.5 log TCID_50_/ml) were assigned a nominal value of 0.0 for mean calculations. Spikes indicate standard errors of the means (SEM). Download FIG S1, TIF file, 2.7 MB. Copyright © 2021 Lorbach et al. 2021 Lorbach et al. https://creativecommons.org/licenses/by/4.0/ This content is distributed under the terms of the Creative Commons Attribution 4.0 International license.

10.1128/mSphere.01170-20.5 FIG S3 Mean change in near-exposed (A) and far-exposed (B) ferret weight from baseline (%) is shown by swine vaccination group exposure. (Inset) Live-attenuated influenza virus (LAIV), killed influenza virus (KV), or sham vaccine (NV). *N* = 3 ferrets per group. Spikes indicate standard errors of the means (SEM). Download FIG S3, TIF file, 2.8 MB. Copyright © 2021 Lorbach et al. 2021 Lorbach et al. https://creativecommons.org/licenses/by/4.0/ This content is distributed under the terms of the Creative Commons Attribution 4.0 International license.

**FIG 4:**
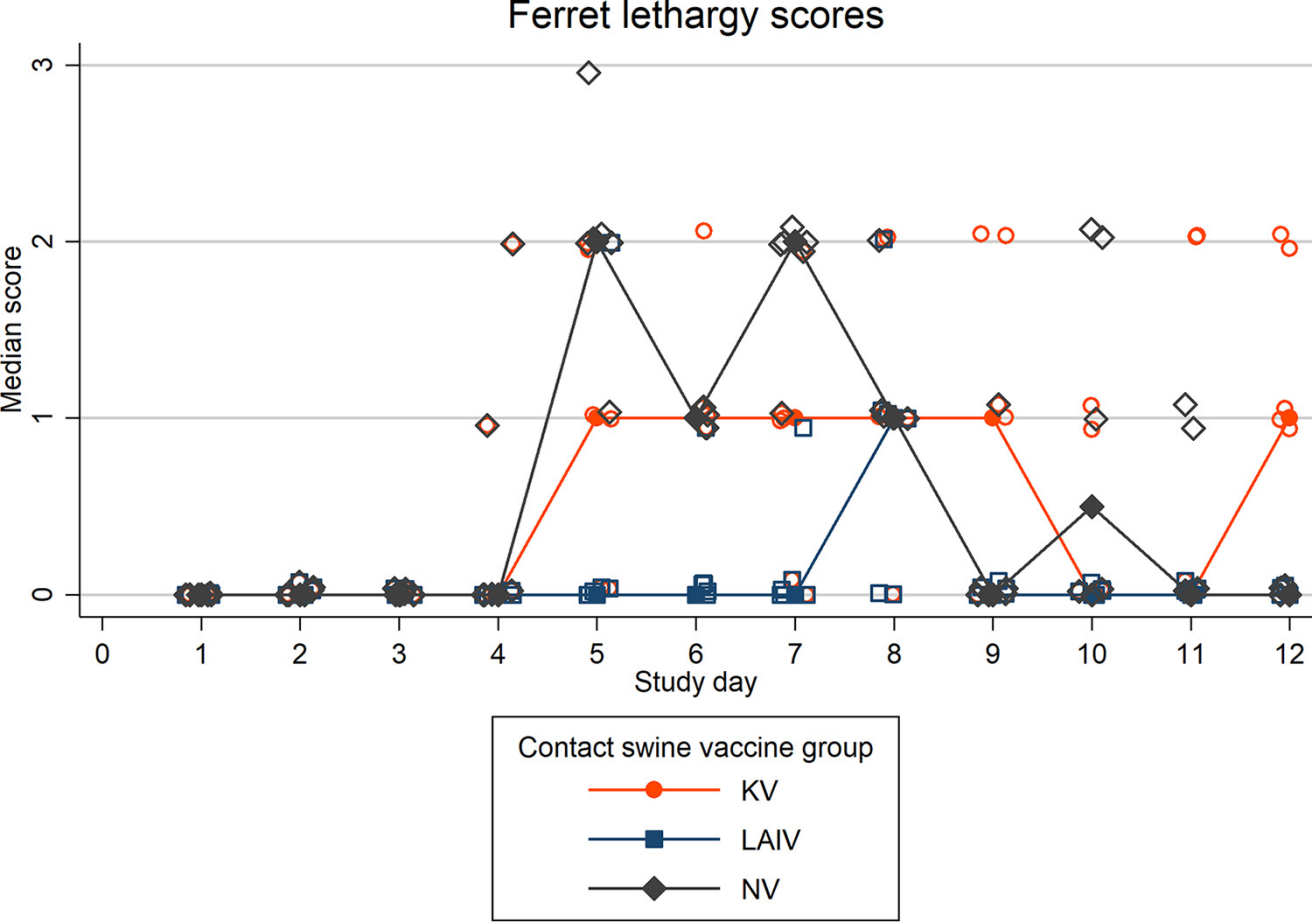
Median ferret lethargy score is shown by swine group exposure: live-attenuated influenza virus (LAIV), killed influenza virus (KV), or sham vaccine (NV). *N* = 6 ferrets per group. Lethargy was scored on an ordinal scale (0, fully playful; 1, responded to playful interaction but did not initiate play; 2, alert but not playful; 3, neither playful nor alert). Median within-group lethargy scores are represented by solid markers (circles, squares, and diamonds) with connecting lines; individual ferret lethargy scores are represented by hollow markers (19).

**FIG 5:**
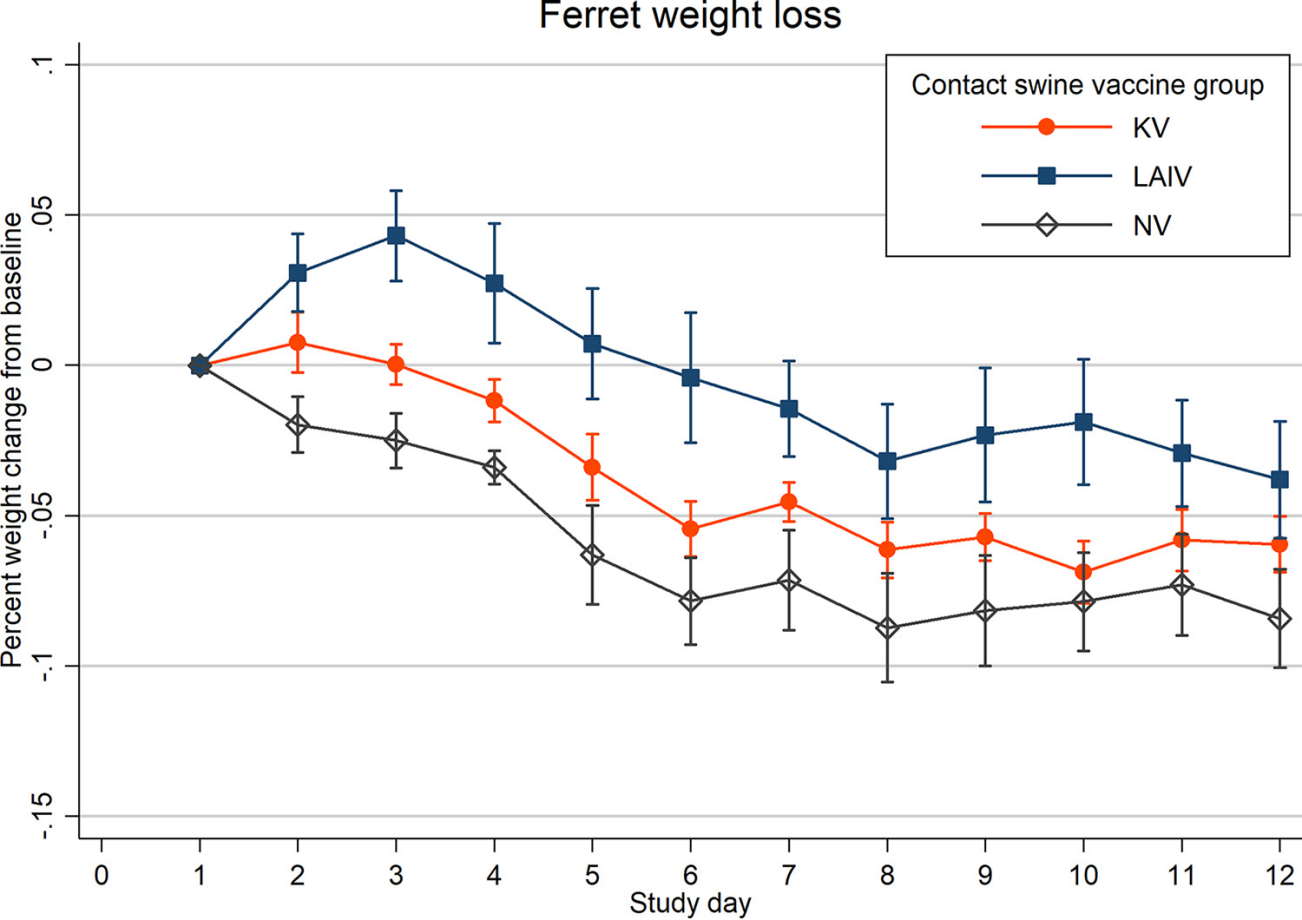
Mean change in ferret weight from baseline (%) is shown by swine group exposure: live-attenuated influenza virus (LAIV), killed influenza virus (KV), or sham vaccine (NV). *N* = 6 ferrets per group. Spikes indicate standard errors of the means (SEM).

**FIG 6:**
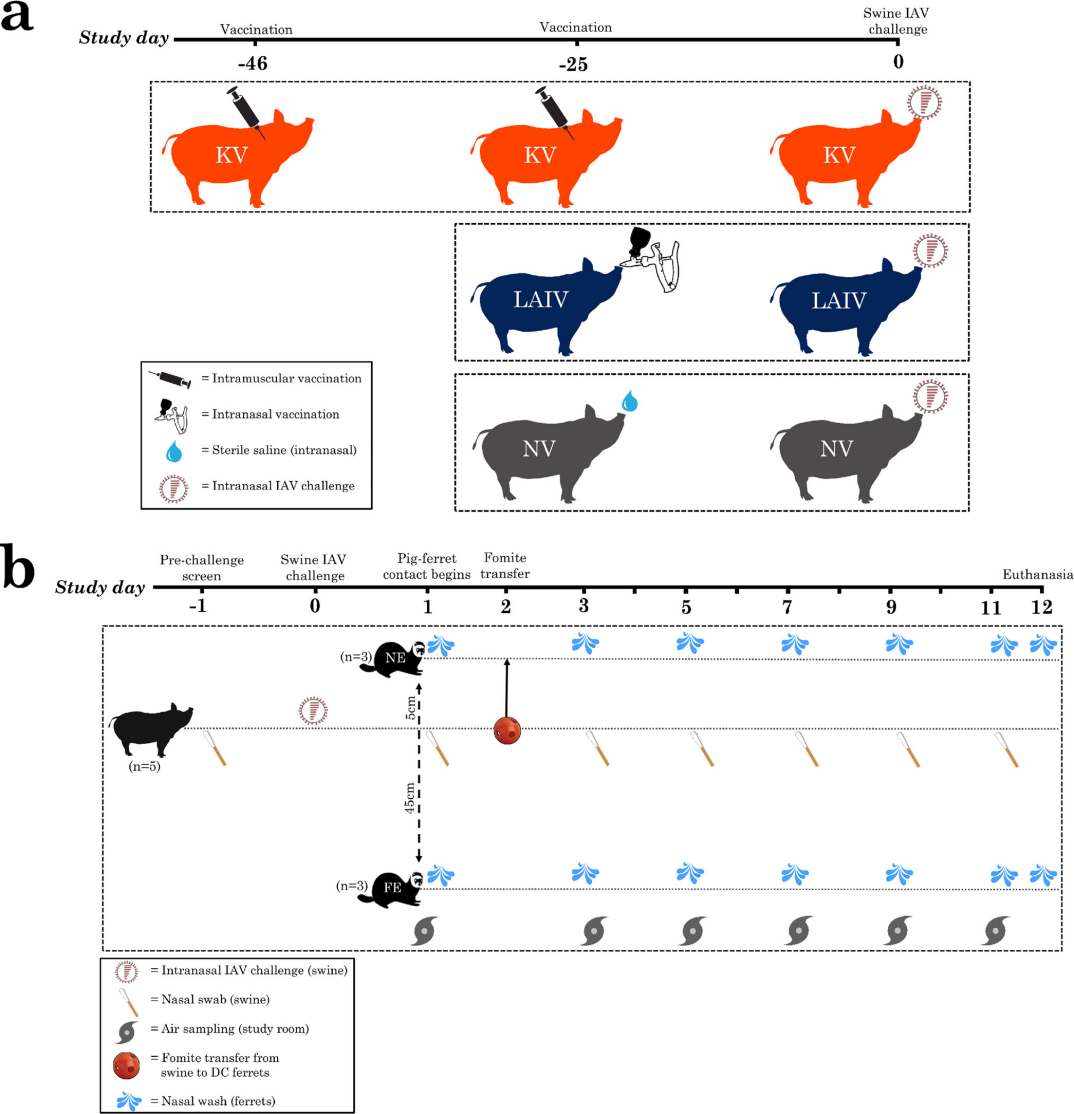
(A) Timing of vaccine treatments for LAIV (live-attenuated influenza virus), KV (killed virus), or NV (sham) swine groups leading up to intranasal challenge with H3N2 subtype IAV (H3 human-like; 3.2010.1). (B) Timeline of animal (pig, near-exposed [NE] ferret, far-exposed [FE] ferret) nasal sample collection, study room air sample collection, and pig-ferret contact occurring for each swine group (LAIV, KV, and NV).

Onset of clinical signs, including nasal discharge, lethargy, and elevated temperatures, was delayed, and severity was reduced in ferrets in the LAIV group compared to ferrets in the NV group (Fig. 4 and 5 and Fig. S2). Onset of nasal signs and lethargy were similar between ferrets in the KV and NV groups, although lethargy was more severe in the NV group (see Fig. 6). Mean weight loss was mild in all three ferret groups (Fig. 5). Weight loss in NV group ferrets began soon after exposure but was delayed by several days in LAIV (NE and FE) and KV (NE) ferret groups (Fig. S3). Mean daily rectal temperatures within the LAIV ferret group gradually increased following exposure, whereas temperatures in the KV and NV groups initially decreased below baseline at 2 dpe and subsequently increased above baseline at 4 dpe (Fig. S2).

10.1128/mSphere.01170-20.4 FIG S2 Median ferret nasal sign scores (A) and mean percent change in ferret rectal temperature from baseline (B) are shown by swine vaccination group exposure. (Inset) Live-attenuated influenza virus (LAIV), killed influenza virus (KV), or sham vaccine (NV). *N* = 6 ferrets per group. Nasal signs observed in individual animals were scored on an ordinal scale (0, no nasal signs; 1, sneezing; 2, nasal discharge on external nares; 3, open mouth breathing) (19). Median within-group clinical sign scores are represented by solid markers (circles, squares, and diamonds) with connecting lines; individual ferret clinical sign scores are represented by hollow markers. Mean percent change in rectal temperature is indicated with a marker, and spikes indicate standard errors of the means (SEM). Download FIG S2, TIF file, 2.7 MB. Copyright © 2021 Lorbach et al. 2021 Lorbach et al. https://creativecommons.org/licenses/by/4.0/ This content is distributed under the terms of the Creative Commons Attribution 4.0 International license.

IAV sequences recovered from nasal washes from all ferrets were >99% identical to the inoculation strain. Upon examination of the hemagglutinin (HA) sequences, a change from alanine to serine at amino acid residue 138 (A138S, H3 numbering) was observed in IAV sequences from one of six LAIV group ferrets, six of six KV group ferrets, and three of six NV group ferrets. Sequencing did not identify any additional known functional mutations.

### Pairwise survival analysis.

The estimated RR obtained from pairwise survival analysis of swine-to-ferret transmission for ferrets exposed to LAIV pigs indicated a significant delay in infection compared with ferrets exposed to NV pigs (RR = 0.66, *P* = 0.028) (Table 1). The estimated odds ratio (OR) for LAIV ferret group compared to NV ferret group was 0.19 (95% confidence interval [CI], 0.04, 0.84), corresponding to an estimated vaccine efficacy (VE) of 81% (95% CI, 16%, 96%) (Table 2). The estimated shape parameter was 4.06 (2.77, 5.60), so the RRs and ORs are quite different (Tables 1 and 2). Point estimates suggest that the KV also delayed the onset of infection in ferrets and that FE ferrets had longer times to infection than NE ferrets (Tables 1 and 2). However, these results were not statistically significant, likely due to small sample size.

**TABLE 1:** Pairwise survival analysis RR with 95% CI and *P* values^*a*^

Comparison	RR	95% CI	*P* value
Vaccination group			
KV versus NV	0.88	0.60, 1.31	0.528
LAIV versus NV	0.66	0.46, 0.95	0.028
Exposure group			
FE versus NE	0.89	0.65, 1.21	0.437

a Abbreviations: NE, near-exposed; FE, far-exposed; KV, killed influenza virus; LAIV, live-attenuated influenza virus; NV, sham vaccine.

**TABLE 2:** Pairwise survival analysis OR and corresponding VE^*a*^

Vaccine	OR	95% CI	VE (%)^*b*^
KV	0.61	0.13, 2.87	39 (−187, 87)
LAIV	0.19	0.04, 0.84	81 (16, 96)

a Abbreviations: KV, killed influenza virus; LAIV, live-attenuated influenza virus.

a Numbers in parentheses are confidence limits for the vaccine efficacy on the odds ratio scale where an odds ratio of 1 corresponds to 0% efficacy and an odds ratio of 0 corresponds to 100% efficacy.

### End-of-study respiratory tract pathology and virus detection.

Microscopic lesions in swine respiratory tissues demonstrated variable chronicity based on inflammatory cell populations and mucosal response to previous damage. Aggregate lung pathology scores were elevated in the KV swine group compared to NV swine (*P* = 0.0088). No significant differences in nasal pathology scores were apparent between groups.

Microscopic lesions in ferret respiratory tissues were relatively acute and primarily consisted of necrotizing bronchiolitis. No significant differences in nasal pathology scores and aggregate lung pathology scores were found between ferret groups.

IAV was not detected in homogenized lung tissue from any pigs in the LAIV or KV groups. Small quantities of viral RNA were detected by PCR in lung tissue from two pigs in the NV group (mean, 3.06 copies/g). Viral RNA was detected in lung specimens from five ferrets in the LAIV group (mean, 48.8 copies/g), two ferrets in the KV group (mean, 5.74 copies/gram), and two ferrets in the NV group (mean, 21.0 copies/gram) at the end of the study. No viable virus was detected from PCR-positive lung tissue homogenates from either species.

## DISCUSSION

Our findings directly support the implementation of preexhibition swine vaccination to protect public health from zoonotic transmission of IAV-S. We have strong evidence that LAIV vaccination of swine delayed infection of ferrets exposed to the pigs following IAV challenge (Tables 1 and 2). However, based on the survival analysis in our study, there is no evidence for the superiority of either LAIV or KV.

Intranasal administration of a high-dose viral inoculum to all swine following vaccination simulated a worst-case scenario wherein multiple pigs are directly exposed to large amounts of infectious IAV immediately prior to prolonged contact with humans. Additionally, we selected an IAV strain that was not represented among the components of either the LAIV or KV vaccine products, which would theoretically maximize the risk of infection of swine and subsequent transmission to ferrets. LAIV and KV products both significantly decreased postchallenge nasal swab peak viral titers and shedding duration in swine (Fig. 1). In general, viral replication was similar in pigs vaccinated with either LAIV or KV. This was corroborated by air sampling data showing the highest levels of IAV in the study room housing NV swine and reduced levels in the LAIV and KV study rooms (Fig. 2). Together, decreased shedding and IAV air levels in the LAIV and KV groups indicate prior use of either vaccine would reduce risk of virus spillover to humans if swine become infected during an exhibition.

No infectious virus was recovered from LAIV or KV pig samples collected at 7, 9, and 12 dpc, but one nasal swab sample collected from a pig in the LAIV group at 11 dpc contained viable virus. This pig may have been reinfected via respiratory droplet transmission from exposed ferrets still shedding IAV immediately prior to that sampling. Alternatively, the animal may have contacted nasal secretions deposited on enclosure surfaces prior to collection of the nasal swab. Because the typical stay of exhibition swine at shows is less than 11 days, the potential impact of this outlier is limited in the field setting.

National health authority recommendations have supported limiting the length of stay for swine at agricultural exhibitions to 72 h or less to minimize potential spread of IAV among show pigs (5, 7). While both the LAIV and KV groups experienced earlier and lower peak shedding, corresponding with overall reductions in cumulative shedding compared to NV pigs, they continued to shed virus for several days beyond 72 h postchallenge. Combining the limited length of stay approach with preexhibition vaccination of swine would further reduce risk of IAV transmission from swine to humans at agricultural exhibitions. Since LAIV and KV pigs experienced reduced cumulative shedding compared to NV pigs, use of either vaccine product would be advisable over no vaccine use in a field setting.

Given the similarities in viral shedding characteristics for the LAIV and KV swine groups, the presence of decreased shedding in ferrets exposed to LAIV pigs compared to ferrets exposed to KV pigs was unexpected (Fig. 1 and 3; see also Fig. S1 in the supplemental material). This trend was present regardless of contact type (NE or FE) between the ferrets and pigs (Fig. S1). We hypothesized postchallenge selection of viral mutants within the swine groups could have led to infection of ferrets with viruses exhibiting altered replication kinetics. The observed serine A138S mutation has been associated with increased infectivity of swine nasal epithelial cell cultures, although additional mutations are required to achieve this phenotype (12). The A138S mutation occurred at different frequencies across the swine treatment groups, and identification of the mutation in ferrets exposed to NV pigs makes it less clear if vaccine use in pigs selected for or against these mutants. The potential impact of vaccine-induced selection pressure on swine-transmitted IAV warrants further investigation.

Altered infectivity of transmitted virus from LAIV swine due to increased mucosal immunity was considered to explain decreased shedding in LAIV group ferrets because LAIV vaccine should have stimulated both serum IgG and mucosal IgA production, whereas KV vaccine should only elicit serum IgG production (13). However, if mucosal immunity were impacting LAIV pig-shed virus infectivity, we would have expected a decrease in LAIV swine viral titers compared to KV swine titers, since *in vitro* viral quantification relied on successful infection of MDCK cells.

Use of either LAIV or KV vaccination in market-age swine was capable of decreasing viral shedding following direct intranasal challenge with a high dose of a contemporary IAV-S associated with zoonotic transmission. Ferrets exposed to LAIV-vaccinated pigs experienced delayed onset to infection, decreased clinical signs, and reduced viral replication, although additional investigation into the underlying mechanism of this apparent benefit is required. Vaccination of exhibition swine should reduce the risk of virus spillover to humans and may prevent emergence of IAV-S with pandemic potential.

## MATERIALS AND METHODS

Ohio State’s Institutional Animal Care and Use and Institutional Biosafety Committees approved protocol no. 2018A00000108 and 2018R00000067, respectively.

### Swine vaccination and challenge.

Fifteen market-age (approximately 6 months of age and weighing >100 kg), male-castrated, mixed-breed domestic pigs from a commercial swine herd known to be free of IAV were assigned to one of three vaccine treatment groups consisting of five pigs each. Swine IAV-naive status was confirmed via serology prior to vaccination using nucleoprotein enzyme-linked immunosorbent assay (see Text S1 in the supplemental material). Vaccine treatment groups were LAIV, KV, and NV. LAIV product virus strains represent two distinct IAV-S clades, H1 gamma2 beta-like (clade 1A.2.3-like) and H3 cluster I (clade 3.1990.1) (14, 15). KV product virus strains represent four distinct IAV-S clades, H1 gamma (clade 1A.3.3.3), H1 delta1 (clade 1B.2.2), H3 cluster IV-A (clade 3.1990.4.1), and H3 cluster IV-B (clade 3.1990.4.2-3) (11). NV swine received a single intranasal vaccination with sterile saline (1.0 ml) and served as the experimental control group. Single-dose (LAIV and NV) and multiple-dose (KV) vaccination regimens were administered according to the manufacturer’s instructions and timed so pigs were fully vaccinated before IAV challenge (Fig. 6A).

10.1128/mSphere.01170-20.6 TEXT S1 Additional details for the methods used in this study. Download Text S1, DOCX file, 0.02 MB. Copyright © 2021 Lorbach et al. 2021 Lorbach et al. https://creativecommons.org/licenses/by/4.0/ This content is distributed under the terms of the Creative Commons Attribution 4.0 International license.

Swine were group-housed in a raised-deck enclosure in a modified biosafety level 2 (BSL-2) facility under constant negative air pressure (Text S1) with an estimated 42 room air changes per hour (study room volume [gross], 81.191 m^3^). One day prior to challenge, swine were confirmed to be IAV negative by nasal swab quantitative reverse transcription-PCR screen and received a single intramuscular injection of 1.0 ml/20 kg of body weight ceftiofur crystalline free acid. Viral challenge was performed at 25 days postvaccination (study day 0). For virus challenge, pigs each received a single, 1.0-ml, intranasal dose (1 × 10^6^ 50% tissue culture infectious dose [TCID_50_]/ml) of A/swine/Ohio/16TOSU4788/2016(H3N2), which is a human-like H3 IAV-S (clade 3.2010.1). This virus shares ≤90% HA segment amino acid identity with component strains in both the LAIV and KV vaccines. The percent HA amino acid differences between H3-subtype vaccine component strains and A/swine/Ohio/16TOSU4788/2016(H3N2) were determined by pairwise alignment of the protein sequences using the MUSCLE alignment program in the Geneious Prime 2021.0.3 software platform: 10.6% for H3 cluster I (clade 3.1990.1) in the LAIV, 12.7% for H3 cluster IV-A (clade 3.1990.4.1), and 12.7% for H3 cluster IV-B (clade 3.1990.4.2-3) in the KV (16). The A/swine/Ohio/16TOSU4788/2016(H3N2) challenge virus was isolated from swine in 2016 and is the same virus as A/Ohio/27/2016(H3N2v), a variant IAV isolated from a human following zoonotic transmission; the latter virus was previously shown to successfully infect naive ferrets and induce mild to moderate clinical disease (17).

### Ferret exposure to swine.

Eighteen male domestic ferrets 4 to 5 months of age were used in this study. Ferrets were confirmed influenza naive by hemagglutination inhibition assay before exposure to challenged swine. Groups of six ferrets were randomly assigned to one of the three swine groups (LAIV, KV, or NV) and introduced to the study room 1 day following swine challenge. Ferrets were individually housed in stainless steel wire cages with either near exposure or far exposure to pigs in the study room. Ferret cages were separated by acrylic barriers to minimize ferret-to-ferret aerosol transmission. Far-exposed (FE; *n* = 3 ferrets) cages were placed with the front of the cage 45 cm from the swine enclosure. Near-exposed (NE; *n* = 3 ferrets) cages were placed with the front of the cage 5 cm from the swine enclosure. All ferrets had access to fabric hammocks for the entire study; however, the NE ferrets had their original hammocks replaced on study day two with hammocks that had been in contact with the pigs to simulate physical contact between NE ferrets and pigs. To accomplish this, hammocks were placed inside Jingle Ball enrichment devices within the swine enclosure for 4 h, during which time the swine played with the devices and deposited nasal secretions on the hammocks. After 4 h, hammocks were removed and placed into the cages of NE ferrets for the duration of the study period.

### Sample collection and animal observation.

Swine nasal swabs, ferret nasal washes, and study room air samples were collected on study days −1 (just swine nasal swab), 1, 3, 5, 7, 9, and 11 and stored at –80°C in sterile brain heart infusion broth; an additional nasal wash sample was collected from ferrets on day 12 (Fig. 6B). Nasal samples from individual pigs were collected using sterile polyester-tipped swabs. Ferret nasal wash samples were collected under sedation. Air samples at each time point were collected over three 15-min intervals using NIOSH air sampling devices placed behind FE ferret cages and connected to a continuous air pump unit (18).

Swine were observed daily to detect the presence of lethargy, nasal discharge, coughing, elevated respiratory rate (>40 breaths per minute), or weight loss, indicated by decreased body condition score. Lethargy and nasal signs in ferrets were assessed using an established scoring system (19). Ferret weights were recorded daily, and rectal temperatures were recorded during nasal wash collection. Animals were humanely euthanized by barbiturate overdose on study day 12, and postmortem tissue collection was performed immediately thereafter.

### Sample processing and analysis.

Swine, ferret, and air samples were processed for IAV detection using quantitative reverse transcription-PCR as previously described (20, 21). To generate an overall concentration of IAV genome copy number per liter of air, quantification data were summed from stage 1, stage 2, and filter-stage air sampling device runs. Virus in PCR-positive samples was quantified by endpoint dilution assay in MDCK cell culture, and final viral titers were determined using the method of Reed and Muench (22). Shedding of infectious virus was indicated by TCID_50_ titers exceeding the assay limit of detection (LOD; 1.5 log TCID_50_/ml). Quantitative nasal and air sample data were log transformed for area under the curve (AUC) analysis. Samples below the LOD were assigned a nominal value of 0.0 log TCID_50_/ml for calculation of mean viral titers and AUC analysis. Descriptive statistics and intergroup comparisons using the two-sample Mann-Whitney test (two sided) was performed with STATA (software version 14.2). A significance threshold of a *P* value of  <0.05 was used for hypothesis testing.

Formalin-fixed tissues were routinely processed and stained with hematoxylin and eosin. A modified semiquantitative scoring approach was devised to assess upper and lower respiratory tract histopathology (23–25) (Text S1).

### Pairwise survival analysis.

Infection and the onset of infectiousness were both defined to be a TCID_50_/ml greater than zero. In each vaccine group, all ferrets were assumed to be at risk of infection from all swine. Ferret-to-ferret pairs were excluded. Each swine-ferret pair was assumed to be at risk of transmission while the pig was exhibiting clinical signs and shedding virus and the ferret was not infected. These pairs were assigned an event indicator equal to one. In each pair, the swine vaccination group (LAIV, KV, or NV) and contact type (NE or FE) were covariates. The reference groups were NV and NE, respectively.

In this data set, there are only two distinct failure times (2 days and 4 days), so we used a parametric regression model (26). We fit the model using exponential, Weibull, and log-logistic failure time distributions and compared them using the Akaike information criterion (AIC). Our results are based on the log-logistic model (AIC, 53.8). We used likelihood ratio confidence intervals for the model coefficients; these are slightly more accurate than Wald confidence intervals in small samples. The log-logistic failure time distribution implies a constant odds ratio (OR) for the probability of infection within any given time interval. With a shape parameter of γ, the rate ratio (RR) is *r* and the OR is *r*^γ^. Because *ρ* depends on multiple parameters, we used the delta method on the log scale to calculate a 95% confidence interval.

### Whole-genome viral sequencing.

IAVs recovered from ferret nasal washes were sequenced to validate that there were no external introductions of non-challenge strain IAV. Reverse transcription and targeted IAV genome amplification were performed with IAV-specific primers (Text S1). The amplicons were purified, followed by quantification of DNA. The DNA library was prepared according to the manufacturer’s protocol and run on the MiSeq platform. Sequences were assembled using IRMA (27). Nucleotide and amino acid sequences were then aligned and visualized using MEGA (28).
