# Supplementary material for: Influenza Vaccination of Swine Reduces Public Health Risk at the Swine-Human Interface
Source: mSphere. 2021 Jun 30;6(3):e01170-20. doi: 10.1128/mSphere.01170-20 (PMC8265676; doi:10.1128/mSphere.01170-20)
Supplement: TABLE S1 [file msphere.01170-20-st001.docx]

**Table S1.** Comparisons of within-group area-under-curve (AUC) estimates of cumulative influenza A virus (IAV) shedding in swine and ferrets.

Abbreviations: KV, killed influenza virus; LAIV, live-attenuated influenza virus; NV, sham

vaccine; TCID, tissue culture infectious dose

^a^ p-values below significance threshold (0.05) are shown in bold
