# Supplementary material for: Influenza Vaccination of Swine Reduces Public Health Risk at the Swine-Human Interface
Source: mSphere. 2021 Jun 30;6(3):e01170-20. doi: 10.1128/mSphere.01170-20 (PMC8265676; doi:10.1128/mSphere.01170-20)
Supplement: TABLE S2 [file msphere.01170-20-st002.docx]

**Table S2.** Mean influenza A virus titers^a^ (tissue culture 50% infectious dose per mL;

TCID_50_/mL) in swine nasal swab and ferret nasal wash samples

|  |  |  | | |  |  | | | | | | | | | | |  |
| --- | --- | --- | --- | --- | --- | --- | --- | --- | --- | --- | --- | --- | --- | --- | --- | --- | --- |
|  |  | Swine, by vaccine group^b^ | | |  | Ferrets, by contact type and swine group exposure^c^ | | | | | | | | | | |  |
|  |  |  | | |  | NE | | |  | FE | | |  | NE and FE | | |  |
| Study day |  | NV | LAIV | KV |  | NV | LAIV | KV |  | NV | LAIV | KV |  | NV | LAIV | KV |  |
| -1 |  | ^d^ | ^d^ | ^d^ |  | - | - | - |  | - | - | - |  | - | - | - |  |
| 0^e^ |  | - | - | - |  | - | - | - |  | - | - | - |  | - | - | - |  |
| 1^f^ |  | 3.29 | 3.09 | 3.10 |  | ^d^ | ^d^ | ^d^ |  | ^d^ | ^d^ | ^d^ |  | ^d^ | ^d^ | ^d^ |  |
| 3 |  | 5.96 | 4.55 | 4.53 |  | 2.39 | 0.00 | 4.11 |  | 3.72 | 0.00 | 0.00 |  | 3.06 | 0.00 | 2.06 |  |
| 5 |  | 6.40 | 2.99 | 2.33 |  | 5.50 | 3.30 | 5.67 |  | 5.56 | 1.56 | 5.56 |  | 5.53 | 2.43 | 5.61 |  |
| 7 |  | 0.78 | 0.00 | 0.00 |  | 4.55 | 1.89 | 4.44 |  | 4.55 | 0.61 | 4.44 |  | 4.55 | 1.25 | 4.44 |  |
| 9 |  | 0.00 | 0.00 | 0.00 |  | 1.11 | 0.00 | 2.06 |  | 0.00 | 2.22 | 2.78 |  | 0.56 | 1.11 | 2.42 |  |
| 11 |  | 0.00 | 0.56 | 0.00 |  | 0.00 | 0.00 | 2.33 |  | 0.00 | 0.00 | 2.39 |  | 0.00 | 0.00 | 2.36 |  |
| 12 |  | - | - | - |  | 0.00 | 0.00 | 1.61 |  | 0.00 | 0.00 | 0.83 |  | 0.00 | 0.00 | 1.22 |  |

Abbreviations: NE, near-exposed; IAV, influenza A virus; FE, far-exposed; KV, killed influenza virus; LAIV, live-attenuated influenza virus; NV, sham vaccine; TCID, tissue culture infectious dose

^a^ PCR-positive samples titered by endpoint dilution (TCID_50_) using the method of Reed and Muench ^23^; samples with IAV titer < assay limit of detection (LOD, 1.5 logTCID_50_/mL) were assigned nominal value of zero for mean calculations

^b^ N=5 pigs per vaccine group

^c^ N=6 ferrets per swine vaccine group, with exposure to pigs via simulated direct contact (NE, n=3) or indirect contact (FE, n=3)

^d^ All samples PCR-negative for IAV

^e^ Swine intranasal challenge with A/swine/Ohio/16TOSU4788/2016 (1x10^6^ TCID_50_)

^f^ Initial ferret exposure to swine. Fomite transfer between pigs and NE ferrets occurred on study day 2.
