# Supplementary figures and images for: Influenza Vaccination of Swine Reduces Public Health Risk at the Swine-Human Interface
Source: mSphere. 2021 Jun 30;6(3):e01170-20. doi: 10.1128/mSphere.01170-20 (PMC8265676; doi:10.1128/mSphere.01170-20)

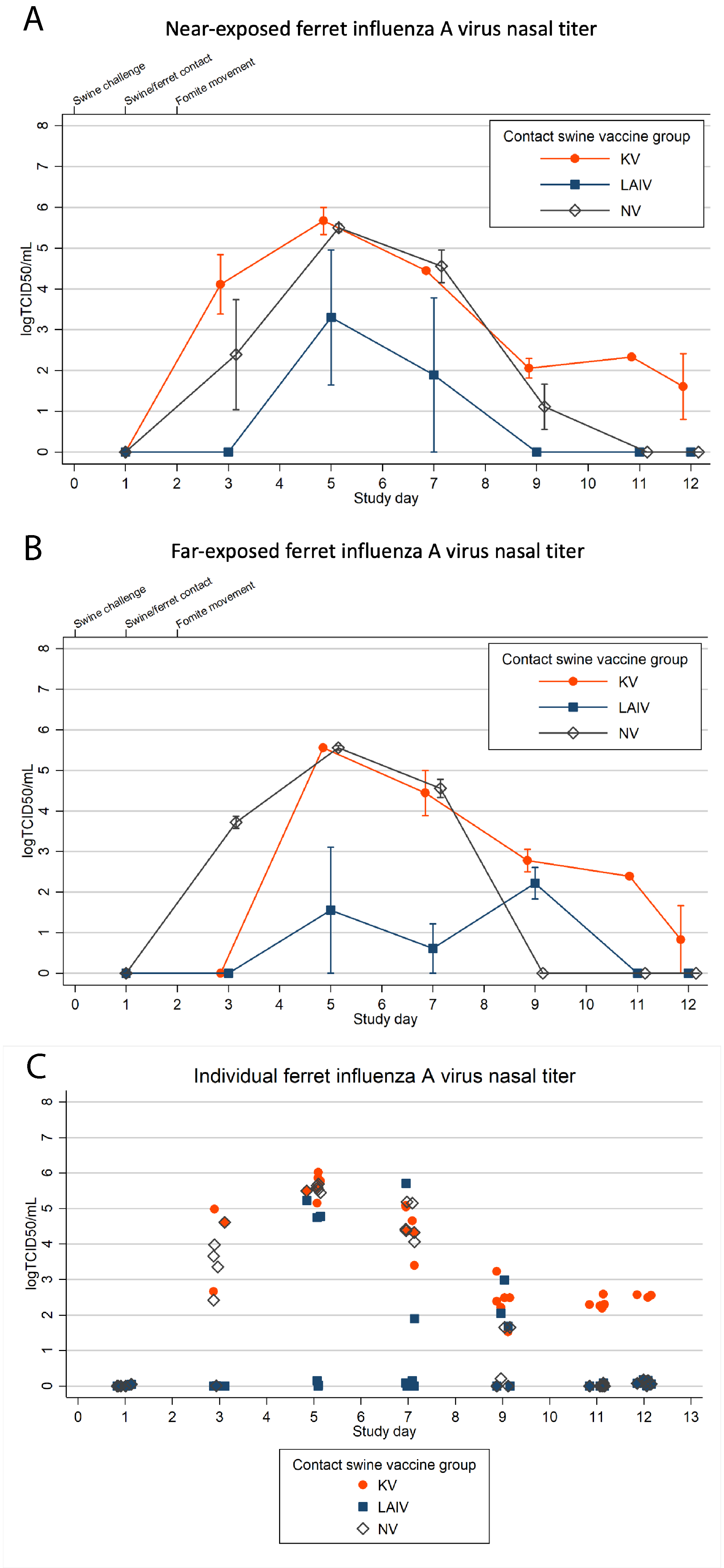

Supplement: FIG S1 [file msphere.01170-20-sf001.tif]

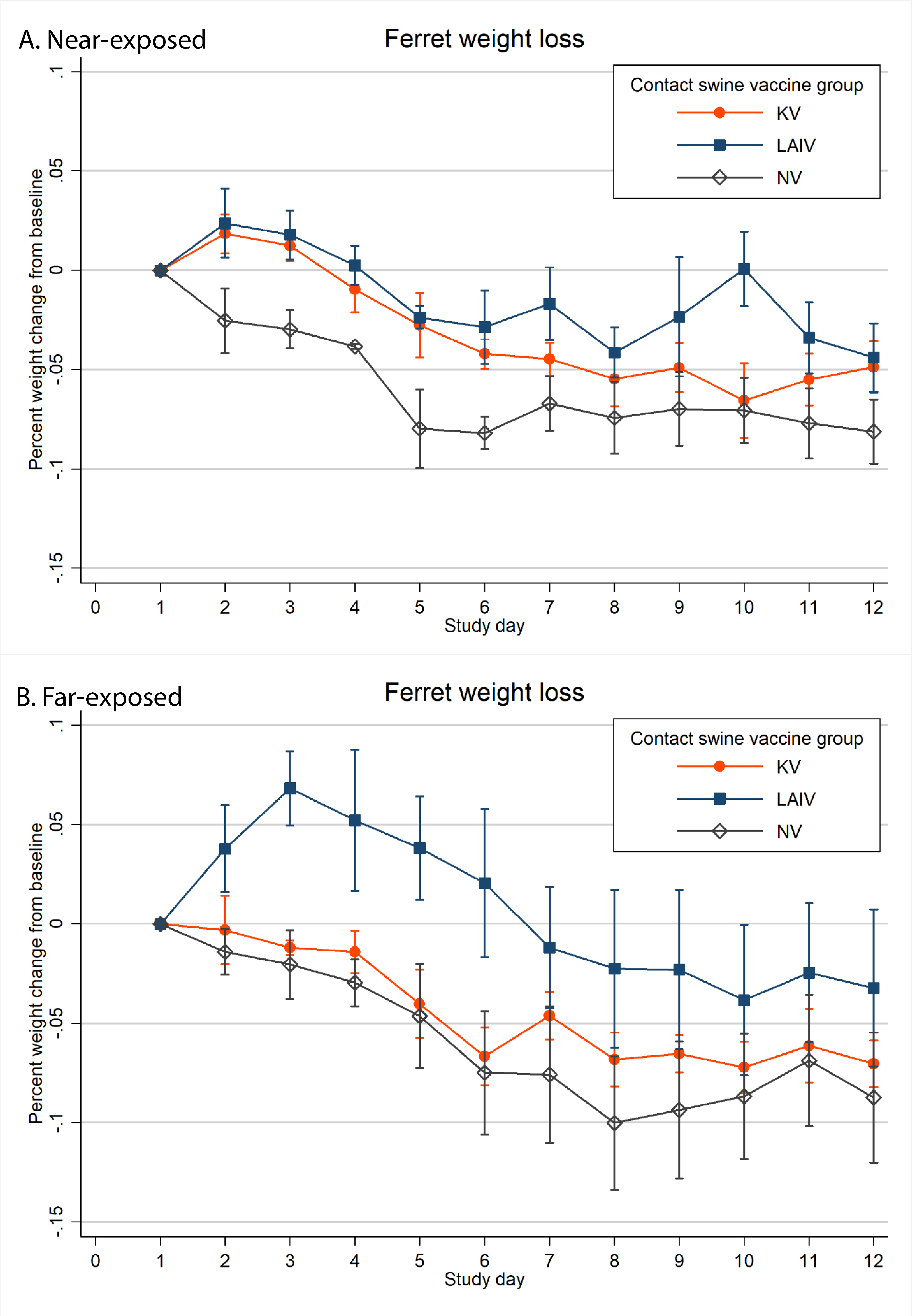

Supplement: FIG S3 [file msphere.01170-20-sf003.tif]

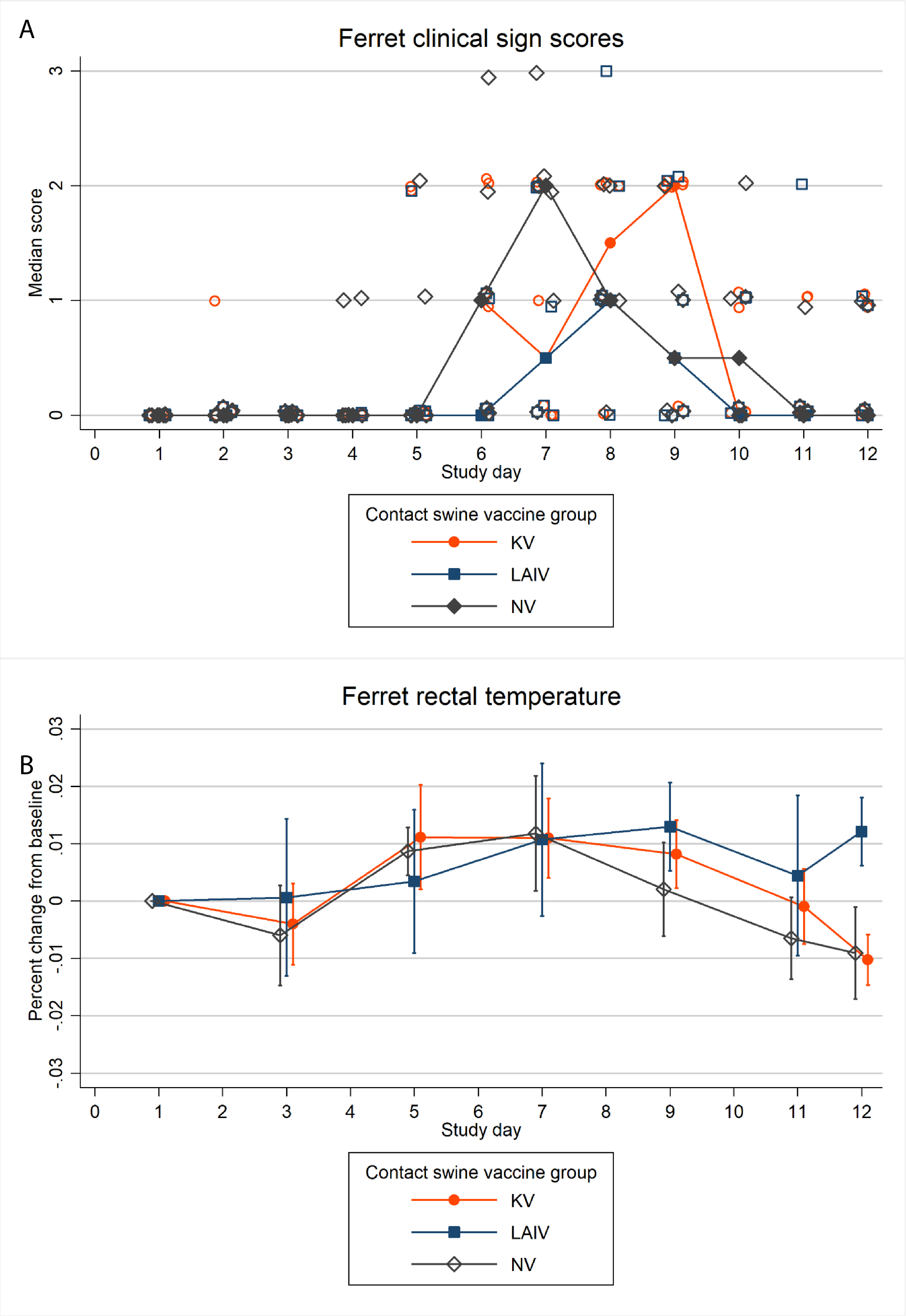

Supplement: FIG S2 [file msphere.01170-20-sf002.tif]
