# Supplementary material for: Influenza Vaccination of Swine Reduces Public Health Risk at the Swine-Human Interface
Source: mSphere. 2021 Jun 30;6(3):e01170-20. doi: 10.1128/mSphere.01170-20 (PMC8265676; doi:10.1128/mSphere.01170-20)
Supplement: TEXT S1 [file msphere.01170-20-s0001.docx]

**Supplemental Methods**

SAMPLE TESTING

- Swine influenza Virus Ab Test, IDEXX, Westbrook, ME
- VetMAX™-Gold SIV Detection Kit (Applied Biosystems, Austin, TX, USA

SEQUENCING

- Superscript™ III One-Step RT-PCR Platinum™ *Taq* Hi-Fi reagents (Life Technologies, Carlsbad, CA, USA) and IAV-specific primers. The amplicons were purified using a MinElute® PCR Purification kit (QIAGEN, Germantown, MD, USA), followed by quantification of DNA using a Qubit® 2.0 Fluorometer. The DNA library was prepared using the Nextera® XT DNA Library Preparation kit (Illumina Inc, San Diego, CA, USA) according to manufacturer’s protocol. Samples were loaded into the Illumina MiSeq™ Reagent Kit V3 cartridge and run on the MiSeq™ Platform (Illumina).
- The sequence of the challenge virus is publicly available in GenBank with sequence accession numbers: KX981507, KX981564, KX981538, KX981496, KX981550, KX981514, KX981489, KX981549 for genome segments 1-8 respectively.

AIR FILTRATION

- HEPA-AIRE® H2KMTF; Abatement Technologies, Suwanee, GA, USA operated at high setting (2,000 CFM)

TREATMENTS USED

- Ceftiofur crystalline free acid: EXCEDE® for Swine, Zoetis Inc., Florham Park, NJ, USA
- Live attenuated influenza virus vaccine: Ingelvac Provenza®, Boehringer Ingelheim Vetmedica, Inc., St. Joseph, MO, USA
- Killed influenza virus vaccine: FluSure XP®, Zoetis Inc., Florham Park, NJ, USA
- Via Prima® Mist sprayers: Item no. 364876, Neogen Corporation, Lansing, MI, USA

VIRUSES FOR HEMAGGLUTINATION INHIBITION ASSAY WITH FERRETS

- A/Singapore/Infimh-16-0019/2016(H3N2)
- A/Slovenia/2903/2015(H1N1)
- B/Colorado/06/2017
- B/Victoria/2/87
- B/Phuket/3073/2013
- B/Yamagata/16/88

FERRETS SUPPLIED BY

- Marshall BioResources, North Rose, NY, USA

FERRET ENRICHMENT DEVICES

- Jingle Ball™, Bio-Serv, Flemington, NJ, USA
